# Supplementary figures and images for: Modelling BK Polyomavirus dissemination and cytopathology using polarized human renal tubule epithelial cells
Source: PLoS Pathog. 2023 Aug 28;19(8):e1011622. doi: 10.1371/journal.ppat.1011622 (PMC10491296; doi:10.1371/journal.ppat.1011622)

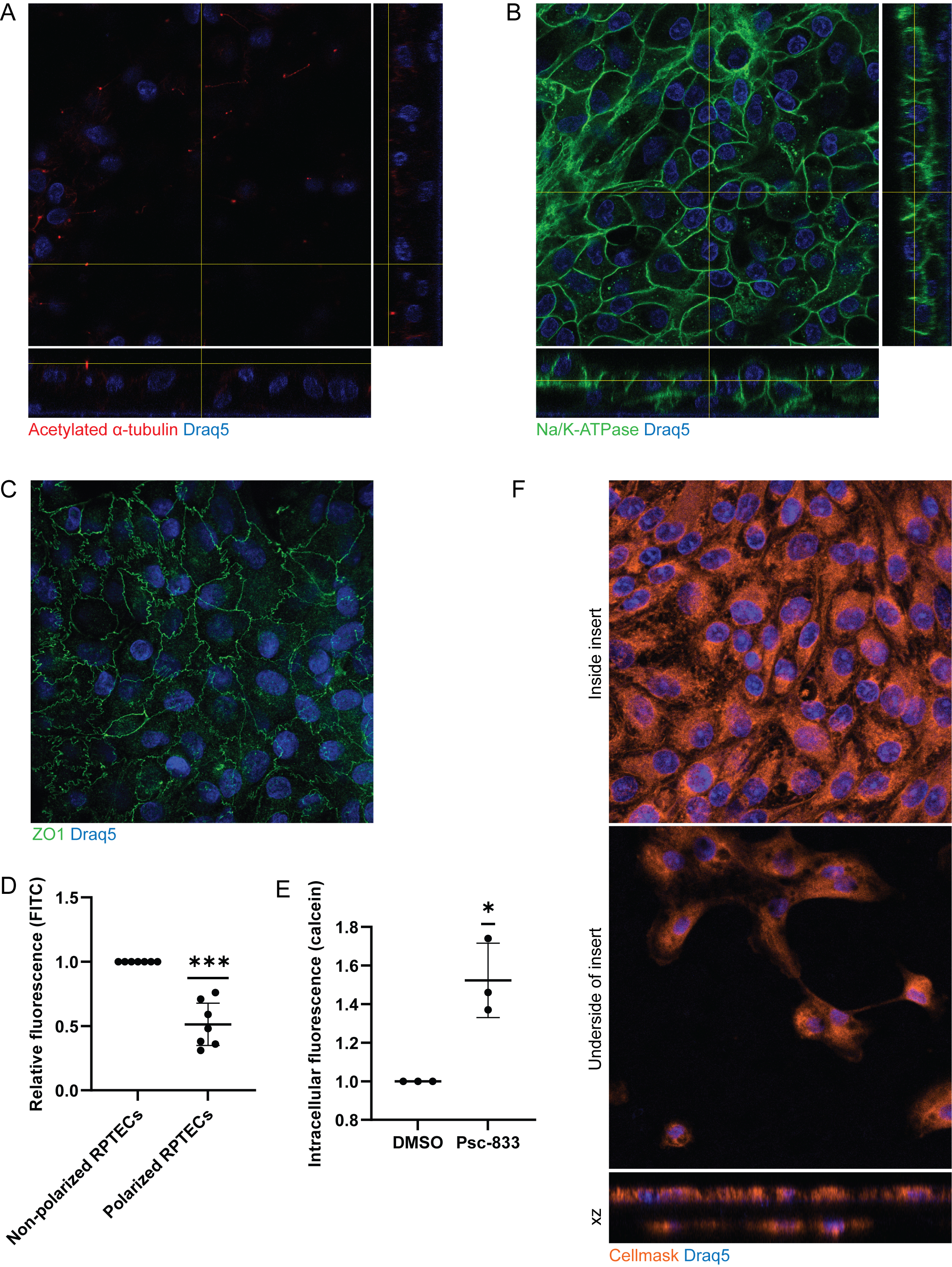

Supplement: S1 Fig — Immunofluorescence staining of RPTECs at 10 dps on Transwell-inserts, pore size 0.4 μm, against markers of apico-basal polarity: (A) Na/K-ATPase (green), (B) acetylated α-tubulin (red) and (C) ZO-1 (green). Nuclei were stained with Draq5 (blue). Images are representative images from at least three independent experiments. (D) Diffusion of FITC-dextran across polarized and non-polarized RPTECs. Data is normalized to the non-polarized control, n = 7 and error bars represent ± SD. *** = P < 0.001, one sample t test. (E) Accumulation of intracellular calcein AM with or without Psc-833, quantified by measuring intracellular fluorescence with a plate reader. Data is normalized to the untreated control. Error bars represent ± SD and n = 3. * = P < 0.05, one sample t test. (F) Confocal microscopy of RPTECs grown on Falcon-inserts with 3.0 μm pore size at 9 dps. Cell membranes were stained with CellMask (orange) while nuclei were stained with Draq5 (blue). Representative images from a z-stack from two independent experiments. (TIF) [file ppat.1011622.s002.tif]

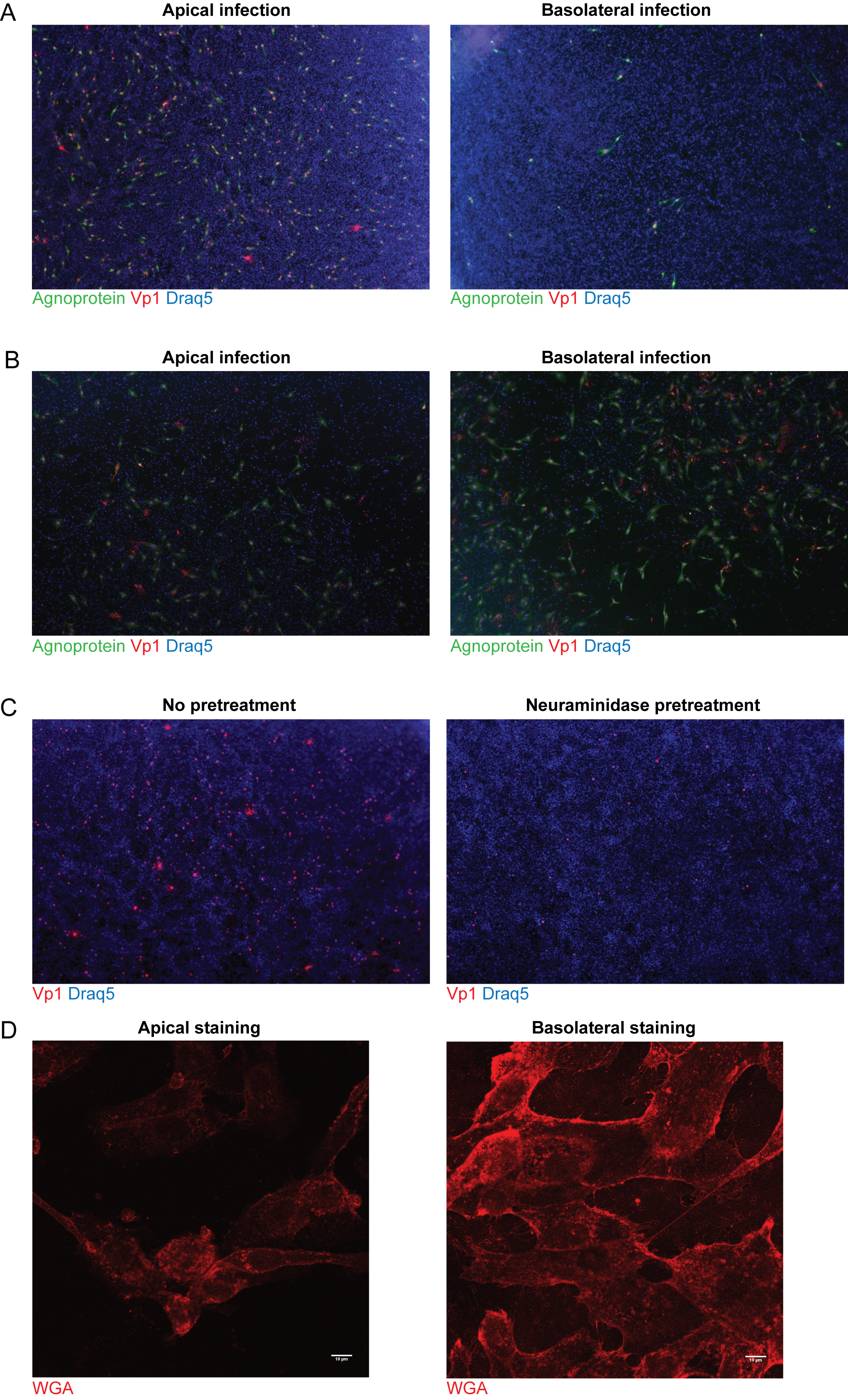

Supplement: S2 Fig — Immunofluorescence staining against agnoprotein (green) and Vp1 (4942) (red) in polarized RPTECs (A) or non-polarized RPTECs (B), infected via the apical or basolateral compartment. (C) Immunofluorescence staining for Vp1 (4942) (red) in polarized RPTECs treated with or without neuraminidase prior to infection. Nuclei were stained with Draq5 in all images. All images are representative images from at least three independent experiments. (D) Confocal microscopy of non-polarized RPTECs stained with Texas Red conjugated wheat germ agglutinin (red) via the apical or basolateral membrane. Scale bar 10 μm. (TIF) [file ppat.1011622.s003.tif]

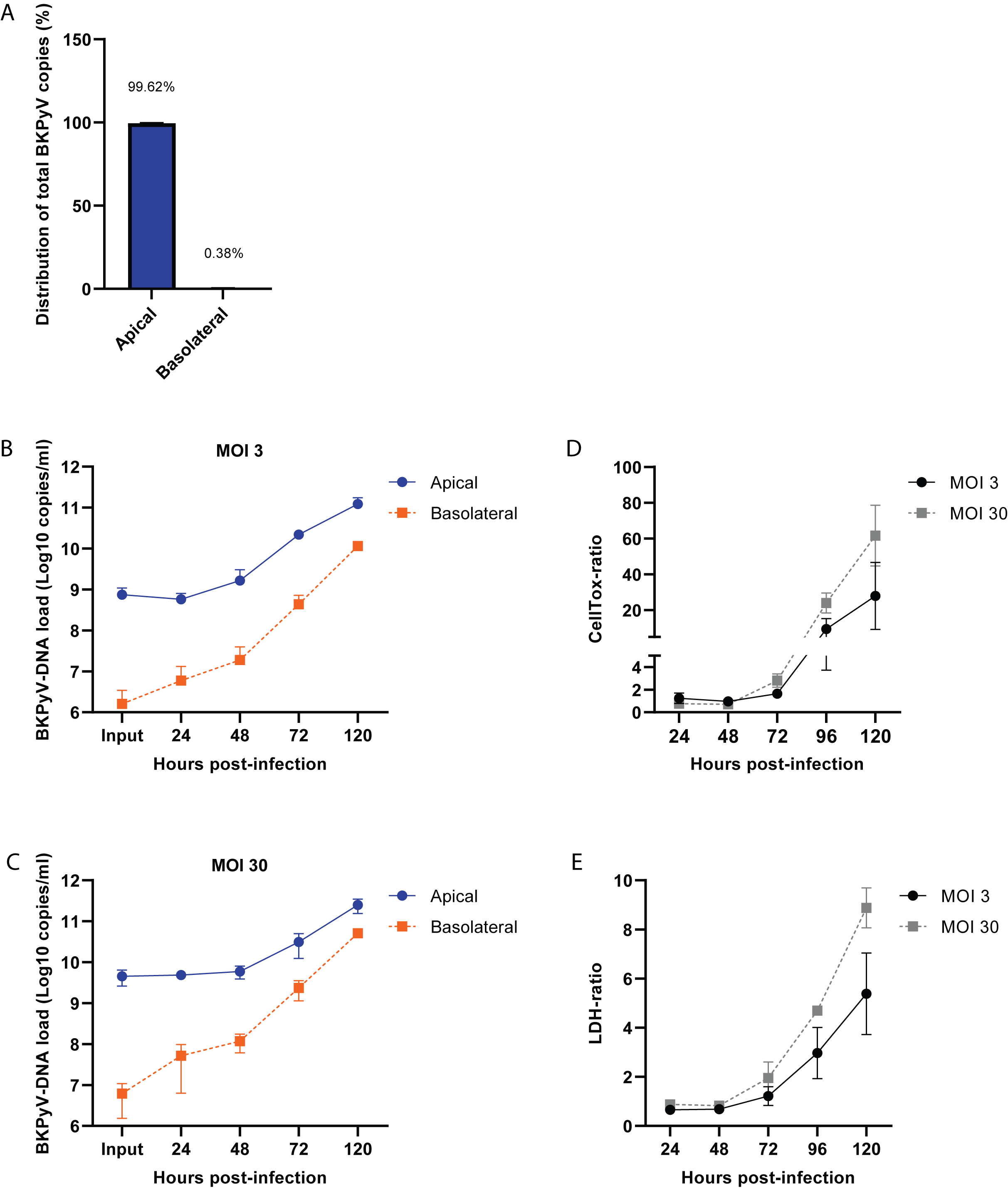

Supplement: S3 Fig — (A) Purified BKPyV was added to the apical compartment. After 2 hours, apical and basolateral supernatants were collected, BKPyV-DNA load (log10 copies/ml) was determined by qPCR and apical and basolateral distribution was calculated. Data was generated from three independent experiments and error bars represent ± SD. (B and C) BKPyV-DNA load (log10 copies/ml) in apical and basolateral supernatants collected from infected polarized RPTECs at indicated timepoints. (B) MOI 3 and (C) MOI 30. Data is generated from three independent experiments and error bars represent ± SD. (D) Widefield microscopy of uninfected and infected (MOI 3 and 30) RPTECs incubated with CellTox dye. Data is presented as CellTox-ratio (mean total fluorescence from infected inserts/mean total fluorescence from mock infected inserts). Error bars represent ± SD and n = 2. (E) Release of LDH into apical supernatants as measured by Promega LDH-Glo Cytotoxicity assay. Data is presented as LDH-ratio (infected-RLU/mock-RLU). Error bars represent ± SD and n = 2. (TIF) [file ppat.1011622.s004.tif]

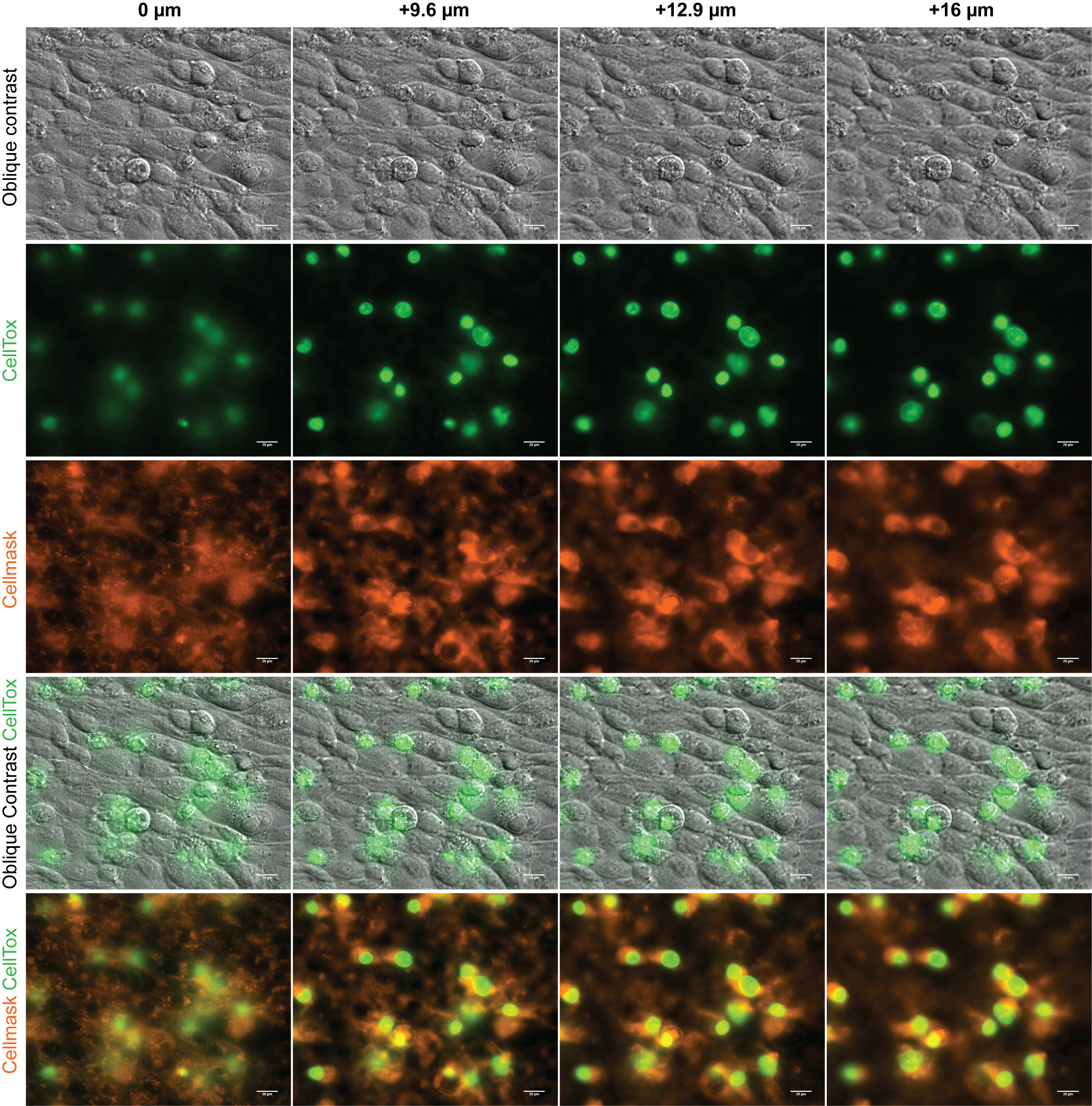

Supplement: S4 Fig — Fully confluent RPTECs were BKPyV infected (MOI 1) and at 4 dpi cells were stained with CellTox dye (green) and CellMask (orange) followed by live-cell imaging and acquisition of z-stacks. Shown is oblique contrast (top row), CellTox (green, second row), CellMask (orange, third row), merge of oblique contrast and CellTox (fourth row) and merge of CellMask and CellTox (bottom row). Each column displays the same z-slice. Images are from a representative z-stack derived from two independent experiments. Scale bar 20 μm. (TIF) [file ppat.1011622.s005.tif]

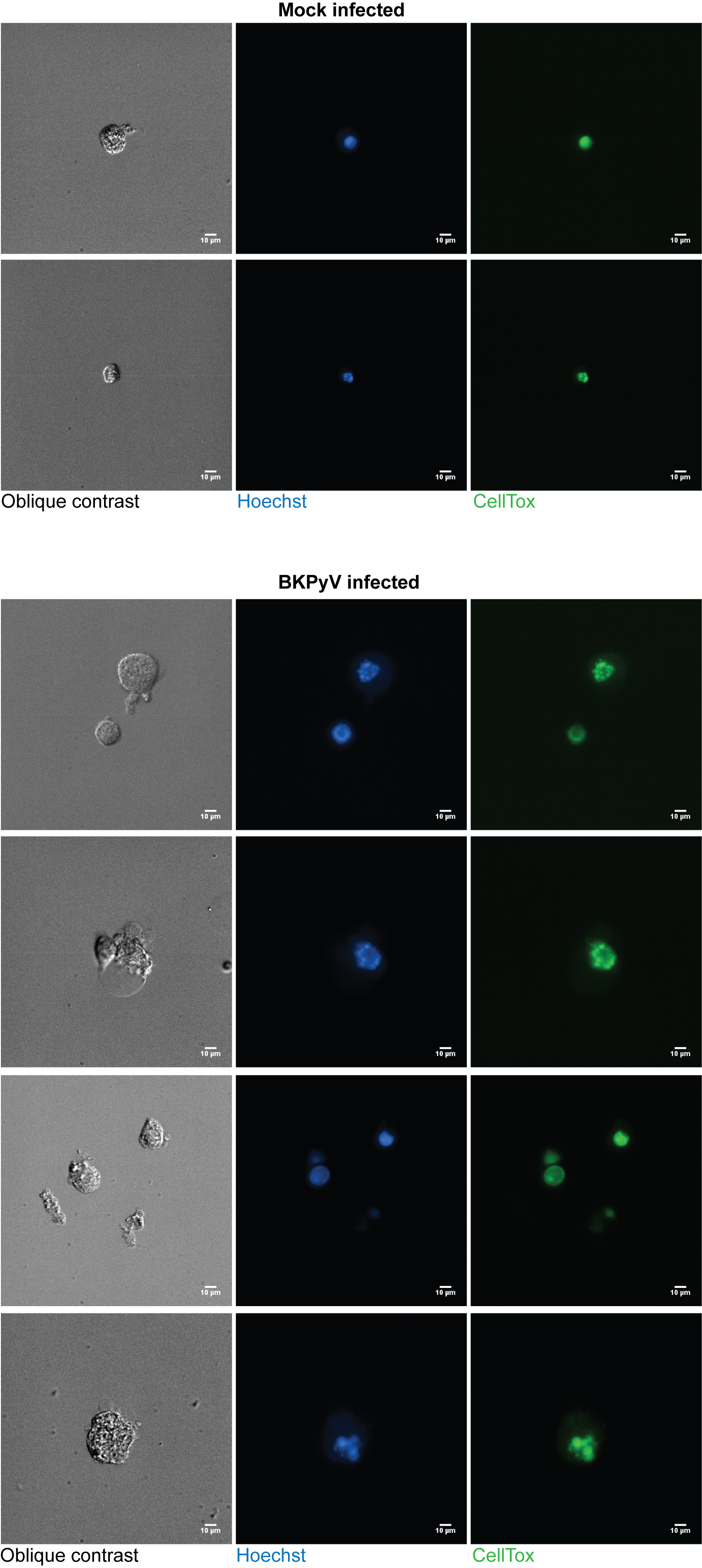

Supplement: S5 Fig — Harvested cells incubated with CellTox dye (green) and Hoechst (blue) and imaged with a combination of oblique contrast and fluorescence microscopy using a Zeiss Celldiscoverer 7 with a 20x objective and 2x tube lens. Images are derived from two independent experiments. Scale bar 10 μm. (TIF) [file ppat.1011622.s006.tif]
