# Supplementary material for: Modelling BK Polyomavirus dissemination and cytopathology using polarized human renal tubule epithelial cells
Source: PLoS Pathog. 2023 Aug 28;19(8):e1011622. doi: 10.1371/journal.ppat.1011622 (PMC10491296; doi:10.1371/journal.ppat.1011622)
Supplement: Attachment — Submitted filename: Responses to review comments Lorentzen et al.pdf [file ppat.1011622.s011.pdf]

Responses to review comments and description of changes

Part I - Summary

Please use this section to discuss strengths/weaknesses of study, novelty/significance, general execution and scholarship.

Reviewer #1: BKPyV primarily infects renal tubular cells and has the potential to destroy the kidney in immunosuppressed individuals; this infection is particularly detrimental in renal transplant recipients. It is currently assumed that intrarenal dissemination of the virus occurs by continuity in renal tubules, however, a detail understanding of this process is not known.

In this study the BKPyV infection is evaluated in a polarized renal proximal tubule epithelial cell line that has the potential to most closely resemble the infectious process in renal tubules.

The study is novel and has experimental/methodological value. Furthermore, conclusions from this study help better understand the lag between the earlier stages of infection and the onset of clinical manifestations.

We thank the reviewer for the positive comments.

Reviewer #2: In this manuscript (PPATHOGENS-D-23-01211), Lorentzen et al describe a new cultured cell model system to study infection by the pathogenic human polyomavirus, BKPyV. BKV infection is widespread in the population and causes severe disease in immunosuppressed individuals, such as renal transplant recipients and patients undergoing bone marrow or stem cell transplants. BKV normally replicates in polarized renal tubule epithelial cells, but in vitro studies are performed in monolayer cell cultures. A few years ago, the Imperiale lab described BKV replication in cultured non-polarized renal proximal tubule epithelial cells (RPTECs). Here, the authors establish polarized cultures of RPTECs on permeable inserts, show BKV replication and cytopathic effects in infected cells, and that the virus preferentially infects and is released from the apical surface (as is the case for the closely related primate polyomavirus, SV40, which also replicates preferentially in kidney), until very late in infection. Virus is also contained in extruded cells with maintenance of epithelial integrity. The work appears competently done and this is likely to be a valuable model system to study this virus.

We thank the reviewer for the positive comments and the thorough assessment of our study.

However, the current work is largely descriptive, and they have not yet used the system to provide new mechanistic insights into BKV replication or pathogenesis.

We agree with the reviewer that our work is mainly descriptive. Nevertheless, we believe that it provides new important insight into three important aspects of BKPyV dissemination and pathogenesis.

1. We demonstrate for the first time that entry of BKPyV mainly occurs via the apical membrane and that this is likely caused by a polarized distribution of sialic acids. Even though apical entry has previously been shown for SV40, detection of this for BKPyV in authentic host cells is valuable. Although the viruses are similar, they are not identical. The DNA sequence identity is about 69% and they were recently found to encode different T antigens splice forms i.e. 17kT and truncT, respectively (Nomburg et al 2022, PloS Pathog.18(4): e1010401). Moreover, they partly infect different cells, altogether suggesting at least some differences in their replication cycle.
2. We provide new insight on how cytopathic effects and cell death influence dissemination of BKPyV. Firstly, by demonstrating minimal diffusion of BKPyV across intact renal proximal tubule epithelium, the leakiest part of the nephron, and barrier conserving apical extrusion of BKPyV infected tubule epithelial cells, we provide new insight on why BKPyV is mainly found in the urine. The above results are also supported by our finding of preferential apical entry. Secondly, we show that eventually widespread cell death disrupts the epithelial barrier and thereby causes leakage of BKPyV and BKPyV-DNA from the apical compartment into the basolateral compartment, providing new insight on how BKPyV DNA gets into the plasma of kidney transplant patients with high-level BKPyV replication.
3. Our findings of apical entry and release into the apical compartment, the latter in the form of extracellular virus and intracellular virus in decoy cells, strongly support that BKPyV disseminate in the tubular fluid along the tubular system to infect new cells downstream in the tubular system via the apical membrane.

Part II – Major Issues: Key Experiments Required for Acceptance
Please use this section to detail the key new experiments or modifications of existing experiments that should be <u>absolutely</u> required to validate study conclusions.

Generally, there should be no more than 3 such required experiments or major modifications

for a "Major Revision" recommendation. If more than 3 experiments are necessary to validate the study conclusions, then you are encouraged to recommend "Reject".

Reviewer #1: None.

We thank the reviewer for the positive assessment of our experimental work.

Reviewer #2: 1. This manuscript would be greatly strengthened if they use their system to address unanswered questions regarding BKV replication or pathogenesis.

We thank the reviewer for this comment. We now plan to use our model to study the innate immune response and the secretome of BKPyV infected cells. The results will hopefully deepen our understanding of BKPyV replication and pathogenesis. However, these projects represent separate studies and was not the scope of this study.

2. Their model for how BKV spreads in vivo is intriguing and consistent with their in vitro studies, but not directly addressed in the experiments.

We thank the reviewer for this comment. We agree that our *in vitro* experiments do not provide definite evidence for our proposed model of BKPyV spread. If an animal model had been available, the spread of BKPyV in the reno-urinary tract, a much more complex system consisting of multiple cell types including immune cells, could have been studied. Despite limitations, we believe our *in vitro* findings provide compelling evidence that support dissemination of BKPyV along the tubular system. That our results harmonize with observations in patients, strengthen our results and proposed model of spread.

We have now included a paragraph on the lack of an animal model in the discussion (line 359-363). Moreover, we have underlined that our model reflects dissemination in the renal tubules and not the complete organism (line 365).

Part III – Minor Issues: Editorial and Data Presentation Modifications

Please use this section for editorial suggestions as well as relatively minor modifications of existing data that would enhance clarity.

Reviewer #1: - In the materials and methods, immunofluorescence staining and microscopy section, a list of antibodies is presented without further clarification. Some of these antibodies are never mentioned in the text and are only mentioned in the figure legends. Since evaluation of the polarized renal tubular cell line is relatively novel, it would be useful if the authors could briefly explain the purpose of the stains.

We thank the reviewer for this suggestion. In the results section, we have explained the target of each of the antibodies used to characterize the RPTECs. For the detection of BKPyV LTag and Vp1 we used two different antibodies for each. For clarity, we have now specified the antibody used in the figure legends and in the methods section.

For example, separate stains for characterization of the viral infection from the stains done to characterize the tubular cell itself (ZO-1, tubulin, etc).

We thank the reviewer for the good suggestion. We have now separated the antibodies into one list of antibodies against BKPyV proteins and one list of antibodies against cellular proteins that were used to characterize the polarized RPTECs.

There is a typo in the last line of page 16 (pylon should be pelvis)

We thank the reviewer for pointing this out and have corrected the error.

Reviewer #2: They should define “decoy cells”.

We thank the reviewer for the suggestion. We have now included a definition of decoy cells in the results section under the headline «BKPyV replication leads to extensive cell detachment». Furthermore, we have stated the purpose of the Papanicolaou staining.

The use of the word “sank” on line 210 is unclear.

We thank the reviewer for pointing this out and have changed “sank” to “decreased”.

Additional changes made to the manuscript:

- We have added two new references and cited reference 38 and 39 one additional time. The edited references are highlighted in yellow.
- The description of the quantitation of Vp1-staining was only described in the figure legend. We have now described it in more detail in the Materials and Methods section.
- We have edited some sentences for increased readability and clarity.
- We have fixed typos
